# High-Density Lipoprotein Particles and Torque Teno Virus in Stable Outpatient Kidney Transplant Recipients

**DOI:** 10.3390/v16010143

**Published:** 2024-01-18

**Authors:** Jip Jonker, Caecilia S. E. Doorenbos, Daan Kremer, Edmund J. Gore, Hubert G. M. Niesters, Coretta van Leer-Buter, Philippe Bourgeois, Margery A. Connelly, Robin P. F. Dullaart, Stefan P. Berger, Jan-Stephan F. Sanders, Stephan J. L. Bakker

**Affiliations:** 1 Department of Internal Medicine, Division of Nephrology, University Medical Center Groningen, University of Groningen, Hanzeplein 1, 9713 GZ Groningen, The Netherlands; 2 Department of Medical Microbiology and Infection Prevention, Division of Clinical Virology, University Medical Center Groningen, University of Groningen, Hanzeplein 1, 9713 GZ Groningen, The Netherlands; 3 bioMérieux, Parc Technologique Delta Sud, 09340 Verniolle, France; 4 Labcorp, Diagnostics Research & Development, Morrisville, NC 27560, USA; 5 Department of Internal Medicine, Division of Endocrinology, University Medical Center Groningen, University of Groningen, Hanzeplein 1, 9713 GZ Groningen, The Netherlands

**Keywords:** torque teno virus, kidney transplantation, immunosuppression, immune system, high-density lipoprotein

## Abstract

Torque teno virus (TTV) is emerging as a potential marker for monitoring immune status. In transplant recipients who are immunosuppressed, higher TTV DNA loads are observed than in healthy individuals. TTV load measurement may aid in optimizing immunosuppressive medication dosing in solid organ transplant recipients. Additionally, there is a growing interest in the role of HDL particles in immune function; therefore, assessment of both HDL concentrations and TTV load may be of interest in transplant recipients. The objective of this study was to analyze TTV loads and HDL parameters in serum samples collected at least one year post-transplantation from 656 stable outpatient kidney transplant recipients (KTRs), enrolled in the TransplantLines Food and Nutrition Cohort (Groningen, the Netherlands). Plasma HDL particles and subfractions were measured using nuclear magnetic resonance spectroscopy. Serum TTV load was measured using a quantitative real-time polymerase chain reaction. Associations between HDL parameters and TTV load were examined using univariable and multivariable linear regression. The median age was 54.6 [IQR: 44.6 to 63.1] years, 43.3% were female, the mean eGFR was 52.5 (±20.6) mL/min/1.73 m^2^ and the median allograft vintage was 5.4 [IQR: 2.0 to 12.0] years. A total of 539 participants (82.2%) had a detectable TTV load with a mean TTV load of 3.04 (±1.53) log10 copies/mL, the mean total HDL particle concentration was 19.7 (±3.4) μmol/L, and the mean HDL size was 9.1 (±0.5) nm. The univariable linear regression revealed a negative association between total HDL particle concentration and TTV load (st.β = −0.17, 95% CI st.β: −0.26 to −0.09, *p* < 0.001). An effect modification of smoking behavior influencing the association between HDL particle concentration and TTV load was observed (P_interaction_ = 0.024). After adjustment for age, sex, alcohol intake, hemoglobin, eGFR, donor age, allograft vintage and the use of calcineurin inhibitors, the negative association between HDL particle concentration and TTV load remained statistically significant in the non-smoking population (st.β = −0.14, 95% CI st.β: −0.23 to −0.04, *p* = 0.006). Furthermore, an association between small HDL particle concentration and TTV load was found (st.β = −0.12, 95% CI st.β: −0.22 to −0.02, *p* = 0.017). Higher HDL particle concentrations were associated with a lower TTV load in kidney transplant recipients, potentially indicative of a higher immune function. Interventional studies are needed to provide causal evidence on the effects of HDL on the immune system.

## 1. Introduction

Torque teno virus (TTV) has recently gained attention as a functional marker of the immune system. TTV is considered ubiquitous in the healthy population and has not been linked to the etiology of clinical disease [1,2,3,4]. TTV load increases with immunosuppression and therefore is a potential marker of immune status, wherein a decline in immune function coincides with a rise in TTV load [1,5,6]. 

In solid organ transplant recipients, a high TTV load is associated with a higher risk of infection, while a low TTV load is associated with a higher risk of rejection of the allograft [7,8,9]. Currently, multiple multicenter intervention trials (TTVguideIT [10], VIGILung [11] and TAOIST) are ongoing to investigate how the TTV-guided dosing of calcineurin inhibitors compares to conventional drug dosage in solid organ transplant recipients. Since TTV load holds the promise of filling the existing gap for a marker that enables personalized immunosuppressive therapy, it has been suggested that TTV load measurements will obtain a place in routine clinical practice regarding the treatment of patients with a solid organ transplant [1,12]. Consequently, action should be taken to discover and describe factors that influence TTV load alongside the immunosuppression state itself. One of these factors might be circulating high-density lipoprotein (HDL) particles. 

Traditionally, the main function of HDL was thought to be that of carrying cholesterol from peripheral tissues to the liver and to thereby antagonize the process of atherosclerosis [13]. An inverse relation between HDL cholesterol (HDL-C) concentration and the risk of cardiovascular disease has indeed been known for several decades [14]. However, more recent research suggests that the function of HDL may be more related to HDL functional properties than to HDL-C concentration [15,16,17]. Indeed, the risk of cardiovascular disease is more closely related to the ability of HDL to promote cellular cholesterol efflux and its anti-inflammatory function and its ability to promote phospholipid efflux than to HDL-C concentration [18,19,20,21]. Furthermore, the total HDL particle concentration may be more strongly associated with the risk of cardiovascular disease than HDL-C [22]. 

In recent years, several other functions besides carrying cholesterol have been attributed to HDL particles. HDL particles are increasingly regarded as both a carrier and a reservoir of a myriad of different molecules [23]. Anti-inflammatory, vasodilatory and antioxidative activities have been attributed to HDL particles [22,24,25,26,27]. Among the molecular cargo of HDL particles are, for example, complement and immunoglobulin proteins [28]. Notably, HDL particles are highly heterogeneous in size and lipid composition [29]. 

Zhang et al. divided murine HDL particles into three HDL subclasses based on size (small (sHDL), medium (mHDL) and large (lHDL)) and found that sHDL particles have a relatively high protein content, with many of these proteins being involved in immune function [28]. Proteins that were predominantly expressed within murine sHDL particles included seven types of immunoglobulins and eight complement proteins [28]. These observations suggest a possible role for sHDL particles in modulating overall immune function. This concept of HDL particles playing a role in immune function is strengthened by the findings of the Copenhagen General Population Study, where lower concentrations of sHDL and mHDL particles were associated with an increased risk of infectious disease morbidity and infectious-disease-related mortality [30]. 

Accordingly, it is plausible that sHDL and mHDL particle concentrations are positively associated with overall immune function due to a role in carrying important immune-related proteins. Concordantly, we hypothesized that sHDL and mHDL are inversely associated with TTV load in kidney transplant recipients and that these associations are stronger than the associations of HDL-C with TTV load. In the current study, this hypothesis was investigated in a large cohort of stable outpatient kidney transplant recipients. 

## 2. Materials and Methods

### 2.1. Study Population

This cross-sectional analysis was performed using the data of kidney transplant recipients (KTRs) participating in the prospective Transplantlines Food and Nutrition Biobank and Cohort study (ClinicalTrials.gov Identifier: NCT02811835). The cohort has been described in several other articles [31,32]. Between November 2008 and March 2011, 817 KTRs with a functioning allograft ≥1 year after transplantation were invited to the outpatient clinic at the University Medical Center Groningen (UMCG). The study was approved by the Institutional Review Board (METc 2008/186). Standard immunosuppressive therapy was given to all KTRs. Participants were excluded if they had a history of alcohol and/or drug addiction, had insufficient knowledge of the Dutch language or were transplanted in a hospital other than the UMCG. A total of 707 KTRs gave written informed consent to participate in the study. A visual representation of the selection process can be found in Supplementary Figure S1. A total of 40 participants without a measured TTV load and 10 participants without measured HDL particle subspecies concentrations due to missing biomaterial were excluded, and 1 participant with a combined kidney–pancreas transplantation was excluded, leaving 656 participants eligible. 

### 2.2. Collection of Data

Blood samples were taken after an overnight fast of 8–12 h and were stored at −80 °C until analyses. The measurement of the TTV load in this cohort has previously been described [31]. An EMAG^®^ Nucleic Acid Extraction System (bioMérieux, Marcy-l’Étoile, France) was used to extract DNA from thawed serum. TTV load was measured by using a TTV R-GENE^®^ (bioMérieux, Marcy-l’Étoile, France) real-time PCR assay on an Applied Biosystems 7500 (Thermo Fisher, Waltham, MA, USA). TTV R-GENE^®^ is a commercially available assay, and all measurements were performed according to the manufacturer’s instructions [33]. Quality control was provided by a spiked control sample with a known quantity of TTV. This was included in each batch of TTV PCR measurements, along with a calibration control for the quantification of TTV. An extraction control was included in each sample. Serum was chosen for measuring TTV load due to the availability of the sample; no significant differences in the cycle threshold (Ct) values were found in a control experiment where measuring the TTV load in serum was compared with that in EDTA plasma. A Ct value of 40 was regarded as the limit of detection. All participants with a TTV load below the limit of detection were labeled as TTV-negative participants. TTV load is presented as log10 copies/mL. 

Lipoprotein particles were measured in EDTA plasma using nuclear magnetic resonance (NMR) spectroscopy with a Vantera^®^ Clinical Analyzer (Labcorp, Morrisville, NC, USA) [34,35]. Further details regarding this analysis have been published elsewhere [36]. Small, medium and large HDL particles were quantified using the amplitudes of their spectroscopically distinct methyl group NMR signals [35]. The total HDL particle concentration was calculated by the sums of the concentrations of small, medium and large HDL particles. The mean HDL size was calculated using the weighted averages derived from the sum of the diameters of small, medium and large HDL particles multiplied by its relative mass percentage. The intra-assay coefficients of variation for the total HDL particle concentration is around 2.0% and amounts to 6.6–10% for the various HDL subfractions [37]. The size range of the total HDL particle concentration was 7.4–13.0 nm, which was divided into small (7.4–8.0 nm), medium (8.1–9.5 nm) and large (9.6–13.0 nm). 

The estimated glomerular filtration rate (eGFR) was measured using the creatinine-based CKD-EPI formula [38]. Data on smoking behavior and alcohol intake were obtained using questionnaires, and the number of alcoholic beverages consumed per week was reported by participants and converted to grams of alcohol per day. 

### 2.3. Statistical Analysis 

Analyses were conducted using “R” statistical software version 4.3.1 [39], and scatterplots were created using the ggplot2 package in “R” [40]. The normality of continuous baseline characteristics was tested via a visual inspection of a histogram. Continuous variables are described as mean with standard deviation (±SD) if normally distributed or median with interquartile range [IQR] if not, and categorical variables are described as count (percentage). If data regarding a characteristic were unavailable for three or more participants, this is noted beneath the table. In all analyses, significance was declared when the two-sided *p*-value was less than 0.05.

For the primary analyses, comparisons of characteristics between participants with a positive TTV PCR and those with a negative TTV PCR were conducted using a *t*-test for normally distributed continuous variables, the Kruskal–Wallis test for non-normally distributed continuous variables or a Chi-Square test for categorical variables. Additionally, univariable linear regression analyses were executed, with each characteristic serving as an independent variable and TTV load as the dependent variable. Only participants exhibiting a positive TTV PCR were included in these regression analyses. The results of the regression analyses are expressed as standardized regression coefficients (st.β), indicating the number of SDs that the dependent variable increases for each SD increase (for continuous) or unit increase (for categorical) in the independent variable. This presentation allows for comparisons of the magnitudes of the associations with the dependent variable across the independent variables. Missing data were managed through listwise exclusion. 

For the secondary analyses, multivariable linear regression analyses were conducted, encompassing all characteristics that exhibited significant associations in the univariable linear regression as independent variables while maintaining TTV load as the dependent variable. As in the primary analyses, only participants exhibiting a positive TTV PCR were included in these regression analyses. Characteristics known to influence TTV load (calcineurin inhibitor usage, age, sex, alcohol consumption and cigarette smoking) were also included in the multivariable linear regression [41,42,43,44,45]. All combinations of independent variables that had a significant association with TTV load in one of the multivariable linear regression analyses were tested for interaction, which was adjusted for usage of calcineurin inhibitors. Multivariable linear regression analyses were repeated with HDL-C instead of the tHDL particle concentration. Furthermore, in participants using cyclosporin, multivariable analyses were repeated, substituting cyclosporin level (a continuous variable) in place of cyclosporin usage (a categorical variable). To mitigate potential issues arising from collinearity, separate multivariable analyses were performed for the total HDL (tHDL) particle concentration and proportions and concentrations of HDL subspecies (sHDL, mHDL and lHDL). Standardized residuals were plotted against the standardized predicted value in all multivariable regression analyses to check for homoscedasticity. The variance inflation factor (VIF) was measured for each independent variable in a multivariable regression analysis [46]. Sensitivity analyses were performed, wherein the participants with the 2.5% highest and 2.5% lowest TTV load and the participants with the 2.5% highest and 2.5% lowest tHDL concentration were excluded. 

## 3. Results

### 3.1. Primary Outcomes

A total of 539 (82.2%) participants of the 656 KTRs had a detectable TTV load. The characteristics of the participants are shown in Table 1. Compared with those who tested negative for TTV, the participants who tested positive for TTV were older (55.7 [45.9, 63.5] vs. 48.9 [39.8, 60.3] years, *p* < 0.001), had a lower eGFR (51.4 (±20.0) vs. 57.4 (±22.3) mL/min/1.73 m^2^, *p* = 0.005), had a shorter allograft vintage (5.10 [1.72, 11.42] vs. 7.05 [4.08, 12.35] years, *p* = 0.009) and had a different calcineurin inhibitor regimen (*p* < 0.001). There was a near statistically significant difference in the proportion of female participants between the TTV-negative group and the TTV-positive group (51.3% vs. 41.6%, *p* = 0.054). The mean tHDL particle concentration showed a near statistically significant difference between the two groups (20.21 (±3.50) µmol/L in the TTV-negative group vs. 19.56 (±3.41) µmol/L in the TTV-positive group, *p* = 0.064). The mean HDL particle size was not different between the two groups (9.13 (±0.50) nm in the TTV-negative group vs. 9.11 (±0.46) nm in the TTV-positive group, *p* = 0.608).

The results from the univariable linear regression analyses are also shown in Table 1. The characteristics with a significant negative association with TTV load were current cigarette smoking, an average daily alcohol intake of >10 g, tHDL particle concentration (st.β = −0.17, 95% CI st.β: −0.26 to −0.09, *p* < 0.001), sHDL particle concentration (st.β = −0.16, 95% CI st.β: −0.24 to −0.07, *p* < 0.001), hemoglobin concentration, eGFR and allograft vintage. Age of the donor, usage of tacrolimus and usage of cyclosporin showed a significant positive association with TTV load. HDL-C was not significantly associated with TTV in the univariable analysis (st.β = −0.07, 95% CI st.β: −0.16 to 0.01, *p* = 0.092). 

### 3.2. Secondary Outcomes

**Figure 1:**
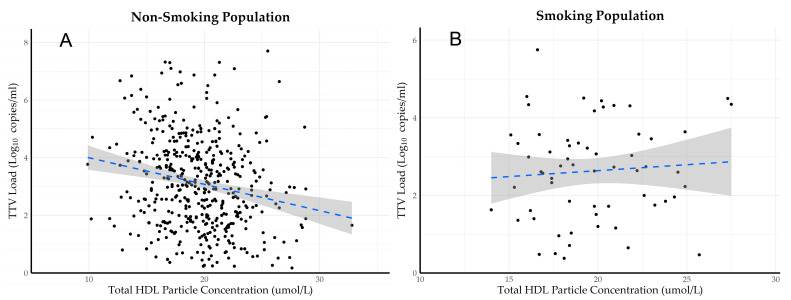
Scatterplots showing associations between tHDL particle concentration and TTV load in the non-smoking population (**A**) and smoking population (**B**). The blue dotted line is the regression line with the gray area representing the 95% CI of the estimate.

The multivariable linear regression analysis (Supplementary Table S1) showed that the negative association between tHDL particle concentration and TTV load was independent of age, sex, current cigarette smoking, average daily alcohol intake >10 g, hemoglobin concentration, eGFR, age of the donor, allograft vintage and usage of a calcineurin inhibitor (st.β = −0.10, 95% CI st.β: −0.19 to −0.01, *p* = 0.024). The analyses of the interaction revealed a noteworthy effect modification, wherein smoking behavior influenced the association between tHDL particle concentration and TTV (P_interaction_ = 0.024). The participants who smoked cigarettes did not exhibit the same negative relationship between tHDL and TTV load as observed in non-smokers, as can be seen in Figure 1. Therefore, further multivariable linear regression analyses were performed with the data from the participants who were not current smokers. Excluding the current smokers strengthened the negative association between tHDL and TTV load, and it remained present independent of adjustment for the previously mentioned characteristics (st.β = −0.14, 95% CI st.β: −0.23 to −0.04, *p* = 0.006), as can be seen in Table 2 (model 1). 

When performing a multivariable regression analysis with cyclosporin level (a continuous variable) instead of cyclosporin usage (a categorical variable), tHDL particle concentration and TTV load were also negatively associated (st.β = −0.20, 95% CI st.β: −0.35 to −0.05, *p* = 0.009), as can be seen in Table 2 (model 3). In contrast to tHDL particle concentrations, HDL-C was not associated with TTV load in the multivariable linear regression analyses (Table 2, models 2/4). 

Multivariable linear regression analyses with the concentrations of the HDL subspecies (Supplementary Table S2) revealed that sHDL concentration was associated with TTV load after adjustment for the characteristics of age, sex, average daily alcohol intake >10 g, hemoglobin concentration, eGFR, age of the donor, allograft vintage and usage of a calcineurin inhibitor (st.β = −0.12, 95% CI st.β: −0.22 to −0.02, *p* = 0.017), while mHDL and lHDL concentrations were not significantly associated. The other multivariable linear regression analyses, with the proportions of HDL particle subspecies as independent variables instead of their concentrations (Supplementary Table S3, models 10/11/12), revealed no significant associations between the proportions of HDL particle subspecies and TTV load, independent of adjustment for the aforementioned characteristics. In the sensitivity analysis with the exclusion of the highest and lowest TTV loads and HDL concentrations (Supplementary Table S3, model 13), the negative association between tHDL particle concentration and TTV load remained statistically significant (st.β = −0.14, 95% CI st.β: −0.24 to −0.04, *p* = 0.008). All independent variables in all multivariable regression analyses had a VIF below 2, indicating that there was no relevant multicollinearity in any of the models. Plotting standardized residuals against the standardized predicted value for all multivariable regression analyses revealed that none of the analyses deviated noticeably from homoscedasticity or normality of the residuals. 

## 4. Discussion

The aim of this article was to investigate whether there is an association between TTV load and HDL particle concentrations in kidney transplant recipients. The current study revealed a consistent negative association between tHDL particle concentration and TTV load in univariable and multivariable regression analyses, whereas HDL-C was not significantly associated with TTV load. A sensitivity analysis confirmed this association. The proportions of HDL particles within specific size windows were not associated with TTV load. The concentration of sHDL particles was negatively associated with TTV load. Taken together, our findings confirm the hypothesis that a higher tHDL and sHDL particle concentration is associated with lower TTV loads, which may represent a better overall immune status, particularly in non-smokers. 

It is plausible that a change in HDL particle concentration would alter immune function and could thereby influence TTV load. A possible mechanism for this influence could be that HDL particles carry proteins that contribute to immune function [28]. The inverse association between TTV load and total HDL particle concentration would fit with the hypothesis that HDL particles are able to modulate immunity. If this hypothesis were to be substantiated, it could be regarded as an argument supporting the TTV-guided dosing of immunosuppressive medication, since this approach to dosing might offer a way to account for the impact of HDL particles on the immune system. The association between TTV load and tHDL particle concentration was only found in the non-smoking participants. This might be linked to the fact that cigarette smoking can modify HDL [47]. It could be theorized that cigarette smoking may attenuate some HDL functionalities, since, for instance, cigarette smoking is known to impair the antioxidative effects of HDL [48]. 

Causality cannot be proven and remains speculative. The function of different HDL particles is largely unknown. It is therefore not possible to rule out that differences in HDL subspecies concentrations could reduce TTV replication instead of lowering TTV load by supporting immune function. Because both immune function and lipid metabolism are associated with a variety of different factors, it is reasonable to speculate that a variable that was not included in this analysis could alter both HDL particle concentration and TTV load. Another possible scenario that could not be dismissed is the potential impact of TTV infection on HDL composition. Inflammation is known to alter HDL composition and function [49]. An ex vivo study revealed that TTV stimulates the production of proinflammatory cytokines [50]. An argument opposing the notion of TTV-mediated inflammation influencing HDL composition is that there is no observed association between TTV load and either leukocyte count or C-reactive protein levels. The hypothesis that HDL particles are beneficial in defense against microorganisms is supported by the fact that the administration of recombinant HDL was found to attenuate the inflammatory effects of administrating Escherichia coli endotoxin in humans [51]. The administration of synthetic apoA-I has also been shown to reduce mortality in rats with induced sepsis [52]. It is, however, unknown whether these beneficial effects of HDL also translate to viruses, as the underlying mechanism of this effect is primarily thought to be that of scavenging endotoxins [23]. 

It remains to be determined whether the association between HDL particle concentration and TTV load exists in healthy individuals or transplant recipients who underwent organ transplantation other than kidney, since it seems likely that there are several ways in which renal disease interacts with HDL or vice versa. Chronic kidney disease (CKD) is associated with lower HDL-C and higher serum triglycerides compared to controls with normal renal function. This effect coincides with a lower HDL phospholipid content and a higher HDL triglyceride content [26]. Interestingly, it is known that, after kidney transplantation, the HDL-C concentration rapidly increases, which is accompanied by a rapid lowering of the triglyceride concentration [53]. After kidney transplantation, lipid metabolism is often altered by immunosuppressive drugs [54]. Furthermore, renal disease seems to alter HDL composition [26,55,56]. 

The participants who tested positive for TTV were older than the participants who tested negative. This difference in TTV positivity by age has previously been described and has been attributed to immunosenescence [41,42]. Sex was not associated with TTV load in both univariable and multivariable analyses, in contrast to earlier findings [41]. Although not all studies found a difference in TTV load between sexes [42,43], the differences in TTV load between sexes could be due to the effects of sex steroid hormones on the immune system [57]. It might be that the effect of sex on TTV load depends on the age category. Haloschan et al. compared differences in TTV load between sexes for different age groups and only found that the average male TTV load was higher than the female TTV load in the age category of 20 to 30 years [57]. Since the 25th percentile of age in this article (45.9 years) is above this age category, it might be that the population is too old to find a significant association between sex and TTV load. It is noteworthy that the proportion of female participants was marginally higher in the TTV-negative group than in the TTV-positive group, approaching statistical significance.

In all analyses, we observed an association of calcineurin inhibitors with TTV load, which is in line with previous research [43,44,58]. Both the use of calcineurin inhibitors and cyclosporin trough levels were associated with TTV load. Furthermore, calcineurin inhibitor users were more likely to have a positive TTV PCR than a negative TTV PCR. This might be a result of the fact that, besides truly TTV-negative participants (with no previous TTV infection), there may also be participants with a negative PCR due to a strong suppression of TTV. No association between BMI and TTV load was found, which is in contrast to a cohort study described by Herz et al., where obesity was associated with a higher TTV load [59]. This discrepancy can possibly be explained by the fact that Herz et al. compared lean participants (BMI 22.3 ± 2.0 kg/m^2^) with obese participants (BMI 40.4 ± 6.6 kg/m^2^), while the participants described in the current article had a rather normal BMI (median BMI 26.1 kg/m^2^ with a 75th percentile of 29.4 kg/m^2^). 

The main limitation of the current study is heterogeneity in immunosuppressive medication and allograft vintage within the study population. As the immune system is an exceptionally intricate function of the human body, a more homogeneous population would be better for testing the hypothesis of this article. Another limitation is the fact that the design of this study is cross-sectional in nature, which precludes the drawing of conclusions on causality. A further limitation is the fact that all participants with a TTV load below the limit of detection were labeled as TTV-negative. In reality, this TTV-negative group most likely consists of participants with above average immune activity/function that suppress TTV well, as well as participants that are negative due to a lack of replication or infection of this virus. In the TTV-negative group, it is conceivable that certain participants may harbor anelloviruses other than TTV. The existing literature indicates that some healthy individuals may test positive for other anelloviruses, such as Torque Teno Mini Virus (TTMV) or Torque Teno Midi Virus (TTMDV) [60]. Another limitation is that not all known factors that influence TTV load were adjusted for in the multivariable linear regression analyses. HIV status, for example, is known to have an association with TTV load, but it was not taken into account in the multivariable analyses [61]. Nevertheless, we do not consider HIV to be prevalent in our overall kidney transplant population. A major strength of this article is the large population.

In conclusion, lower tHDL and sHDL particle concentrations were found to be associated with a higher TTV load in KTRs who did not smoke cigarettes, indicating that differences in HDL particle concentration might impact immune function. Causality can, however, not be concluded from this cross-sectional study. Interventional studies are needed to provide causal evidence on the impact of HDL on the immune system. An example of such an interventional study would involve assessing the impact of administering recombinant HDL on TTV load. The associations of HDL parameters with TTV load may have implications for personalized TTV-guided immunosuppression. Whether and how to consider HDL parameters in the TTV-guided dosing of immunosuppressive medication also remain to be determined.

## Figures and Tables

**Table 1:** Population characteristics. Categorical variables are presented as *n* (%), normally distributed continuous variables as mean (SD) and non-normally distributed continuous variables as median [IQR]. Results from univariable linear regression are shown on the right for each variable, with TTV load as the independent variable; only participants that tested positive for TTV were included in the regression analyses. Data regarding smoking behavior were missing for 36 participants, and data regarding age of the donor were missing for 18 participants.

	TTV-Negative (*n* = 117)	TTV-Positive (*n* = 539)	Univariable Linear Regression in the TTV-Positive Group (TTV Load Is the Dependent Variable)	
			st.β	95% CI st.β
Recipient characteristics				
Age (years) **	48.9 [39.8, 60.3]	55.7 [45.9, 63.5]	0.07	−0.02; 0.15
Female sex	60 (51.3)	224 (41.6)	−0.01	−0.18; 0.16
BMI (kg/m^2^)	26.0 (4.3)	26.8 (4.8)	0.01	−0.08; 0.09
Smoking behavior				
- Never smoked cigarettes	42 (38.9)	217 (42.4)	Reference	
- Stopped smoking cigarettes	48 (44.4)	234 (45.7)	−0.05	−0.24; 0.13
- Current cigarette smoking	18 (16.7)	61 (11.9)	−0.35	−0.63; −0.06
Average daily alcohol intake >10 g	43 (36.8)	172 (31.9)	−0.19	−0.37; −0.01
History of myocardial infarction	3 (2.6)	27 (5.0)	0.06	−0.33; 0.45
History of cerebral vascular accident and/or transient ischemic attack	5 (4.3)	36 (6.7)	0.17	−0.17; 0.51
History of diabetes mellitus	22 (18.8)	133 (24.7)	0.18	−0.02; 0.37
Blood test results				
TTV load (log10 copies/mL)	Not applicable	3.04 (1.53)	Not applicable	
Hemoglobin (mmol/L)	8.22 (1.08)	8.24 (1.06)	−0.14	−0.23; −0.06
Leucocytes (109/L)	8.01 (2.36)	8.17 (2.54)	−0.06	−0.14; 0.03
C-reactive protein (mg/L)	1.40 [0.65, 3.60]	1.60 [0.70, 4.57]	−0.02	−0.11; 0.06
eGFR (mL/min/1.73 m2) *	57.4 (22.3)	51.4 (20.0)	−0.16	−0.25; −0.08
Cholesterol (mmol/L)	5.00 (1.04)	5.15 (1.11)	0.00	−0.09; 0.08
Triglycerides (mmol/L)	1.52 [1.10, 2.33]	1.68 [1.25, 2.24]	−0.02	−0.10; 0.07
LDL cholesterol (mmol/L)	2.86 (0.87)	3.01 (0.93)	0.02	−0.06; 0.11
Total LDL particle concentration (umol/L)	1325 (400)	1384 (408)	0.04	−0.04; 0.13
Mean LDL size (nm)	21.0 [20.6, 21.3]	20.9 [20.5, 21.3]	0.01	−0.08; 0.09
HDL parameters				
HDL cholesterol (mmol/L)	1.30 [1.10, 1.70]	1.30 [1.10, 1.60]	−0.07	−0.16; 0.01
tHDL particle concentration (umol/L)	20.21 (3.50)	19.56 (3.41)	−0.17	−0.26; −0.09
Mean HDL size (nm)	9.13 (0.50)	9.11 (0.46)	0.01	−0.08; 0.09
Distribution of HDL particles (% of tHDL)				
- sHDL	74.4 [65.5, 81.1]	74.6 [65.5, 82.1]	−0.02	−0.11; 0.06
- mHDL	14.0 [9.5, 19.3]	13.4 [8.2, 19.6]	0.00	−0.09; 0.08
- lHDL	9.8 [6.2, 15.8]	10.6 [6.5, 16.0]	0.03	−0.05; 0.12
sHDL particle concentration (umol/L)	14.67 (3.19)	14.26 (3.12)	−0.16	−0.24; −0.07
mHDL particle concentration (umol/L)	2.80 [1.80, 3.80]	2.60 [1.60, 3.90]	−0.04	−0.13; 0.04
lHDL particle concentration (umol/L)	1.90 [1.20, 3.50]	2.00 [1.20, 3.40]	−0.02	−0.11; 0.06
Donor characteristics				
Age (years)	43 [29, 52]	47 [33, 55]	0.09	0.01; 0.18
Living donor	45 (38.5)	184 (34.1)	−0.01	−0.18; 0.17
Allograft vintage (years) *	7.05 [4.08, 12.35]	5.10 [1.72, 11.42]	−0.12	−0.21; −0.04
Pre-emptive transplantation	22 (18.8)	81 (15.0)	0.10	−0.14; 0.33
Medication				
Calcineurin inhibitor usage **				
- Not using a calcineurin inhibitor	75 (64.1)	211 (39.1)	Reference	
- Using tacrolimus	18 (15.4)	97 (18.0)	0.66	0.42; 0.89
- Using cyclosporin	24 (20.5)	231 (42.9)	0.44	0.26; 0.62
Using prednisolone	115 (98.3)	535 (99.3)	−0.13	−1.12; 0.85
Using proliferation inhibitor	104 (88.9)	442 (82.0)	−0.11	−0.34; 0.11
Using statin	58 (49.6)	290 (53.8)	−0.05	−0.22; 0.12

* significant difference between the two groups: *p*-value between 0.01 and 0.001. ** significant difference between the two groups: *p*-value < 0.001.

**Table 2:** Results of multivariable linear regression using, among others, usage of tacrolimus and usage of cyclosporin as independent variables. Continuous variables were standardized (st.β. with 95% confidence intervals).

	Model 1 (R^2^ = 0.12, *n* = 440)			Model 2 (R^2^ = 0.11, *n* = 440)			Model 3 (R^2^ = 0.20, *n* = 180)			Model 4 (R^2^ = 0.18, *n* = 180)		
	st.β	95% CI	*p*-Value	st.β	95% CI	*p*-Value	st.β	95% CI	*p*-Value	st.β	95% CI	*p*-Value
Age of the recipient	0.08	−0.02; 0.17	0.106	0.09	0.00; 0.19	0.052	0.12	−0.03; 0.26	0.107	0.12	−0.02; 0.27	0.099
Female sex	0.03	−0.17; 0.23	0.765	0.01	−0.19; 0.22	0.896	0.03	−0.28; 0.33	0.867	−0.03	−0.34; 0.28	0.846
Average daily alcohol intake >10 g	−0.15	−0.35; 0.06	0.157	−0.18	−0.39; 0.02	0.082	−0.25	−0.59; 0.08	0.136	−0.33	−0.66; 0.00	0.053
Hemoglobin	−0.06	−0.16; 0.05	0.289	−0.06	−0.17; 0.04	0.256	−0.11	−0.27; 0.06	0.200	−0.12	−0.29; 0.04	0.148
eGFR	−0.04	−0.15; 0.06	0.426	−0.06	−0.17; 0.05	0.294	−0.08	−0.24; 0.09	0.359	−0.10	−0.27; 0.06	0.227
Age of the donor	−0.02	−0.13; 0.09	0.677	−0.02	−0.14; 0.09	0.661	−0.09	−0.27; 0.08	0.298	−0.11	−0.29; 0.07	0.229
Allograft vintage	−0.08	−0.19; 0.03	0.138	−0.08	−0.20; 0.03	0.135	−0.06	−0.23; 0.10	0.464	−0.05	−0.22; 0.12	0.556
Calcineurin inhibitor usage												
- Not using a calcineurin inhibitor	Reference			Reference								
- Using cyclosporin	0.31	0.10; 0.52	0.004	0.32	0.10; 0.53	0.004						
- Using tacrolimus	0.60	0.33; 0.88	<0.001	0.63	0.35; 0.91	<0.001						
Cyclosporin trough level							0.32	0.18; 0.47	<0.001	0.31	0.17; 0.46	<0.001
Total HDL particle concentration	−0.14	−0.23; −0.04	0.006				−0.20	−0.35; −0.05	0.009			
HDL cholesterol				−0.08	−0.18; 0.02	0.115				−0.12	−0.27; 0.03	0.116

## Data Availability

Data underlying the results can be made available by contacting the corresponding author.

## Supplementary Materials

The following supporting information can be downloaded at https://www.mdpi.com/article/10.3390/v16010143/s1, Figure S1: flowchart illustrating the participant selection process for this article; Table S1: results of multivariable linear regression; Table S2: results of multivariable linear regression, Table S3: results of multivariable linear regression, Table S4: STROBE Statement—checklist for cross-sectional studies.

## References

[1] Focosi D., Antonelli G., Pistello M., Maggi F. (2016). Torquetenovirus: The human virome from bench to bedside. Clin. Microbiol. Infect..

[2] Focosi D., Spezia P.G., Macera L., Salvadori S., Navarro D., Lanza M., Antonelli G., Pistello M., Maggi F. (2020). Assessment of prevalence and load of torquetenovirus viraemia in a large cohort of healthy blood donors. Clin. Microbiol. Infect..

[3] Spezia P.G., Focosi D., Baj A., Novazzi F., Ferrante F.D., Carletti F., Minosse C., Matusali G., Maggi F. (2023). TTV and other anelloviruses: The astonishingly wide spread of a viral infection. Asp. Mol. Med..

[4] Vasilyev E.V., Trofimov D.Y., Tonevitsky A.G., Ilinsky V.V., Korostin D.O., Rebrikov D.V. (2009). Torque Teno Virus (TTV) distribution in healthy Russian population. Virol. J..

[5] De Vlaminck I., Khush K.K., Strehl C., Kohli B., Luikart H., Neff N.F., Okamoto J., Snyder T.M., Cornfield D.N., Nicolls M.R. (2013). Temporal Response of the Human Virome to Immunosuppression and Antiviral Therapy. Cell.

[6] Studenic P., Bond G., Kerschbaumer A., Bécède M., Pavelka K., Karateev D., Stieger J., Puchner R., Mueller R.B., Puchhammer-Stöckl E. (2022). Torque Teno Virus quantification for monitoring of immunomodulation with biologic compounds in the treatment of rheumatoid arthritis. Rheumatology.

[7] Doberer K., Schiemann M., Strassl R., Haupenthal F., Dermuth F., Görzer I., Eskandary F., Reindl-Schwaighofer R., Kikić Ž., Puchhammer-Stöckl E. (2020). Torque teno virus for risk stratification of graft rejection and infection in kidney transplant recipients—A prospective observational trial. Am. J. Transplant..

[8] Jaksch P., Görzer I., Puchhammer-Stöckl E., Bond G. (2022). Integrated Immunologic Monitoring in Solid Organ Transplantation: The Road Toward Torque Teno Virus-guided Immunosuppression. Transplantation.

[9] van Rijn A.L., Roos R., Dekker F.W., Rotmans J.I., Feltkamp M. (2023). Torque teno virus load as marker of rejection and infection in solid organ transplantation—A systematic review and meta-analysis. Rev. Med. Virol..

[10] Haupenthal F., Rahn J., Maggi F., Gelas F., Bourgeois P., Hugo C., Jilma B., Böhmig G.A., Herkner H., Wolzt M. (2023). A multicentre, patient- and assessor-blinded, non-inferiority, randomised and controlled phase II trial to compare standard and torque teno virus-guided immunosuppression in kidney transplant recipients in the first year after transplantation: TTVguideIT. Trials.

[11] Gottlieb J., Reuss A., Mayer K., Weide K., Schade-Brittinger C., Hoyer S., Jaksch P. (2021). Viral load-guided immunosuppression after lung transplantation (VIGILung)—Study protocol for a randomized controlled trial. Trials.

[12] Elwasif S.M., Denewar A.A., Khreba N., Sheashaa H. (2022). Torque Teno Virus Polymerase Chain Reaction Titer: A Promising Immunometry. Exp. Clin. Transplant..

[13] Stein O., Stein Y. (1999). Atheroprotective mechanisms of HDL. Atherosclerosis.

[14] Castelli W.P., Doyle J.T., Gordon T., Hames C.G., Hjortland M.C., Hulley S.B., Kagan A., Zukel W.J. (1977). HDL cholesterol and other lipids in coronary heart disease. The cooperative lipoprotein phenotyping study. Circulation.

[15] Lincoff A.M., Nicholls S.J., Riesmeyer J.S., Barter P.J., Brewer H.B., Fox K.A.A., Gibson C.M., Granger C., Menon V., Montalescot G. (2017). Evacetrapib and Cardiovascular Outcomes in High-Risk Vascular Disease. New Engl. J. Med..

[16] Kappelle P.J.W.H., van Tol A., Wolffenbuttel B.H.R., Dullaart R.P.F. (2011). Cholesteryl ester transfer protein inhibition in cardiovascular risk management: Ongoing trials will end the confusion. Cardiovasc. Ther..

[17] Mutharasan R.K., Thaxton C.S., Berry J., Daviglus M.L., Yuan C., Sun J., Ayers C., Lloyd-Jones D.M., Wilkins J.T. (2017). HDL efflux capacity, HDL particle size, & high-risk carotid atherosclerosis in a cohort of asymptomatic older adults: The Chicago Healthy Aging Study. J. Lipid Res..

[18] Rohatgi A., Khera A., Berry J.D., Givens E.G., Ayers C.R., Wedin K.E., Neeland I.J., Yuhanna I.S., Rader D.R., de Lemos J.A. (2014). HDL cholesterol efflux capacity and incident cardiovascular events. New Engl. J. Med..

[19] Ebtehaj S., Gruppen E.G., Bakker S.J., Dullaart R.P., Tietge U.J.F. (2019). HDL (High-Density Lipoprotein) Cholesterol Efflux Capacity Is Associated With Incident Cardiovascular Disease in the General Population. Arter. Thromb. Vasc. Biol..

[20] Jia C., Anderson J.L., Gruppen E.G., Lei Y., Bakker S.J., Dullaart R.P.F., Tietge U.J. (2021). High-Density Lipoprotein Anti-Inflammatory Capacity and Incident Cardiovascular Events. Circulation.

[21] Sato M., Neufeld E.B., Playford M.P., Lei Y., Sorokin A.V., Aponte A.M., Freeman L.A., Gordon S.M., Dey A.K., Jeiran K. (2023). Cell-free high-density lipoprotein-specific phospholipid efflux assay predicts incident cardiovascular disease. J. Clin. Investig..

[22] Singh K., Chandra A., Sperry T., Joshi P.H., Khera A., Virani S.S., Ballantyne C.M., Otvos J.D., Dullaart R.P.F., Gruppen E.G. (2020). Associations Between High-Density Lipoprotein Particles and Ischemic Events by Vascular Domain, Sex, and Ethnicity: A Pooled Cohort Analysis. Circulation.

[23] Catapano A.L., Pirillo A., Bonacina F., Norata G.D. (2014). HDL in innate and adaptive immunity. Cardiovasc. Res..

[24] Rached F.H., Chapman M.J., Kontush A. (2015). HDL particle subpopulations: Focus on biological function. BioFactors.

[25] Shah A.S., Tan L., Long J.L., Davidson W.S. (2013). Proteomic diversity of high density lipoproteins: Our emerging understanding of its importance in lipid transport and beyond. J. Lipid Res..

[26] Vaziri N.D. (2016). HDL abnormalities in nephrotic syndrome and chronic kidney disease. Nat. Rev. Nephrol..

[27] Deets A., Joshi P.H., Chandra A., Singh K., Khera A., Virani S.S., Ballantyne C.M., Otvos J.D., Dullaart R.P.F., Gruppen E.G. (2023). Novel Size-Based High-Density Lipoprotein Subspecies and Incident Vascular Events. J. Am. Heart Assoc..

[28] Zhang Y., Gordon S.M., Xi H., Choi S., Paz M.A., Sun R., Yang W., Saredy J., Khan M., Remaley A.T. (2019). HDL subclass proteomic analysis and functional implication of protein dynamic change during HDL maturation. Redox Biol..

[29] Triolo M., Annema W., Dullaart R.P.F., Tietge U.J.F. (2013). Assessing the functional properties of high-density lipoproteins: An emerging concept in cardiovascular research. Biomark. Med..

[30] Harsløf M., Pedersen K.M., Afzal S., Smith G.D., Nordestgaard B.G. (2023). Lower levels of small HDL particles associated with increased infectious disease morbidity and mortality: A population-based cohort study of 30 195 individuals. Cardiovasc. Res..

[31] Gore E.J., Gomes-Neto A.W., Wang L., Bakker S.J.L., Niesters H.G.M., de Joode A.A.E., Verschuuren E.A.M., Westra J., Van Leer-Buter C. (2020). Torquetenovirus Serum Load and Long-Term Outcomes in Renal Transplant Recipients. J. Clin. Med..

[32] van den Berg E., Engberink M.F., Brink E.J., van Baak M.A., Gans R.O.B., Navis G., Bakker S.J.L. (2013). Dietary protein, blood pressure and renal function in renal transplant recipients. Br. J. Nutr..

[33] Kulifaj D., Durgueil-Lariviere B., Meynier F., Munteanu E., Pichon N., Dubé M., Joannes M., Essig M., Hantz S., Barranger C. (2018). Development of a standardized real time PCR for Torque teno viruses (TTV) viral load detection and quantification: A new tool for immune monitoring. J. Clin. Virol..

[34] Matyus S.P., Braun P.J., Wolak-Dinsmore J., Saenger A.K., Jeyarajah E.J., Shalaurova I., Warner S.M., Fischer T.J., Connelly M.A. (2015). HDL particle number measured on the Vantera^®^, the first clinical NMR analyzer. Clin. Biochem..

[35] Jeyarajah E.J., Cromwell W.C., Otvos J.D. (2006). Lipoprotein particle analysis by nuclear magnetic resonance spectroscopy. Clin. Lab. Med..

[36] van den Berg E.H., Flores-Guerrero J.L., Gruppen E.G., Garcia E., Connelly M.A., de Meijer V.E., Bakker S.J.L., Blokzijl H., Dullaart R.P.F. (2022). Profoundly Disturbed Lipoproteins in Cirrhotic Patients: Role of Lipoprotein-Z, a Hepatotoxic LDL-like Lipoprotein. J. Clin. Med..

[37] Garcia E., Bennett D.W., Connelly M.A., Jeyarajah E.J., Warf F.C., Shalaurova I., Matyus S.P., Wolak-Dinsmore J., Oskardmay D.N., Young R.M. (2020). The extended lipid panel assay: A clinically-deployed high-throughput nuclear magnetic resonance method for the simultaneous measurement of lipids and Apolipoprotein B. Lipids Health Dis..

[38] Levey A.S., Stevens L.A., Schmid C.H., Zhang Y.L., Castro A.F., Feldman H.I., Kusek J.W., Eggers P., Van Lente F., Greene T. (2009). A new equation to estimate glomerular filtration rate. Ann. Intern. Med..

[39] R Core Team (2023). R: A Language and Environment for Statistical Computing.

[40] Wickham H. (2016). Ggplot2: Elegant Graphics for Data Analysis.

[41] Giacconi R., Maggi F., Macera L., Spezia P.G., Pistello M., Provinciali M., Piacenza F., Basso A., Bürkle A., Moreno-Villanueva M. (2020). Prevalence and loads of torquetenovirus in the European mark-age study population. J. Gerontol. Ser. A Biol. Sci. Med. Sci..

[42] Westman G., Schoofs C., Ingelsson M., Järhult J.D., Muradrasoli S. (2020). Torque teno virus viral load is related to age, CMV infection and HLA type but not to Alzheimer’s disease. PLoS ONE.

[43] Strassl R., Doberer K., Rasoul-Rockenschaub S., Herkner H., Görzer I., Kläger J.P., Schmidt R., Haslacher H., Schiemann M., A Eskandary F. (2019). Torque Teno Virus for Risk Stratification of Acute Biopsy-Proven Alloreactivity in Kidney Transplant Recipients. J. Infect. Dis..

[44] Jaksch P., Kundi M., Görzer I., Muraközy G., Lambers C., Benazzo A., Hoetzenecker K., Klepetko W., Puchhammer-Stöckl E. (2018). Torque teno virus as a novel biomarker targeting the efficacy of immunosuppression after lung transplantation. J. Infect. Dis..

[45] Doorenbos C.S.E., Jonker J., Hao J., Gore E.J., Kremer D., Knobbe T.J., de Joode A.A.E., Sanders J.S.F., Thaunat O., Niesters H.G.M. (2023). Smoking, Alcohol Intake and Torque Teno Virus in Stable Kidney Transplant Recipients. Viruses.

[46] Thompson C.G., Kim R.S., Aloe A.M., Becker B.J. (2017). Extracting the Variance Inflation Factor and Other Multicollinearity Diagnostics from Typical Regression Results. Basic Appl. Soc. Psychol..

[47] He B.-M., Zhao S.-P., Peng Z.-Y. (2013). Effects of cigarette smoking on HDL quantity and function: Implications for atherosclerosis. J. Cell. Biochem..

[48] Chen H.-Y., Li S.-C., Chen L.-F., Wang W., Wang Y., Yan X.-W. (2019). The effects of cigarette smoking and smoking cessation on high-density lipoprotein functions: Implications for coronary artery disease. Ann. Clin. Biochem. Int. J. Biochem. Lab. Med..

[49] Marsche G., Saemann M.D., Heinemann A., Holzer M. (2013). Inflammation alters HDL composition and function: Implications for HDL-raising therapies. Pharmacol. Ther..

[50] Rocchi J., Ricci V., Albani M., Lanini L., Andreoli E., Macera L., Pistello M., Ceccherini-Nelli L., Bendinelli M., Maggi F. (2009). Torquetenovirus DNA drives proinflammatory cytokines production and secretion by immune cells via toll-like receptor 9. Virology.

[51] Pajkrt D., E Doran J., Koster F., Lerch P.G., Arnet B., van der Poll T., Cate J.W.T., van Deventer S.J. (1996). Antiinflammatory effects of reconstituted high-density lipoprotein during human endotoxemia. J. Exp. Med..

[52] Zhang Z., Datta G., Zhang Y., Miller A.P., Mochon P., Chen Y.-F., Chatham J., Anantharamaiah G.M., White C.R., Constantinou C. (2009). Apolipoprotein A-I mimetic peptide treatment inhibits inflammatory responses and improves survival in septic rats. Am. J. Physiol. Heart Circ. Physiol..

[53] Thompson M., Ray U., Yu R., Hudspeth A., Smillie M., Jordan N., Bartle J. (2016). Kidney Function as a Determinant of HDL and Triglyceride Concentrations in the Australian Population. J. Clin. Med..

[54] Badiou S., Cristol J.-P., Mourad G. (2009). Dyslipidemia following kidney transplantation: Diagnosis and treatment. Curr. Diabetes Rep..

[55] Holzer M., Birner-Gruenberger R., Stojakovic T., El-Gamal D., Binder V., Wadsack C., Heinemann A., Marsche G. (2011). Uremia alters HDL composition and function. J. Am. Soc. Nephrol..

[56] Gliwińska A., Ćwiklińska A., Czaplińska M., Wieczorek-Breitzke E., Kortas-Stempak B., Kuchta A., Dębska-Ślizień A., Król E., Jankowski M. (2023). Changes in the size and electrophoretic mobility of HDL subpopulation particles in chronic kidney disease. J. Nephrol..

[57] Haloschan M., Bettesch R., Görzer I., Weseslindtner L., Kundi M., Puchhammer-Stöckl E. (2014). TTV DNA plasma load and its association with age, gender, and HCMV IgG serostatus in healthy adults. Age.

[58] Görzer I., Haloschan M., Jaksch P., Klepetko W., Puchhammer-Stöckl E. (2014). Plasma DNA levels of Torque teno virus and immunosuppression after lung transplantation. J. Heart Lung Transplant..

[59] Herz C.T., Kulterer O.C., Kulifaj D., Gelas F., Franzke B., Haupenthal F., Prager G., Langer F.B., Marculescu R., Haug A.R. (2022). Obesity is associated with a higher Torque Teno viral load compared to leanness. Front. Endocrinol..

[60] Cebriá-Mendoza M., Beamud B., Andreu-Moreno I., Arbona C., Larrea L., Díaz W., Sanjuán R., Cuevas J.M. (2023). Human Anelloviruses: Influence of Demographic Factors, Recombination, and Worldwide Diversity. Microbiol. Spectr..

[61] Shibayama T., Masuda G., Ajisawa A., Takahashi M., Nishizawa T., Tsuda F., Okamoto H. (2001). Inverse relationship between the titre of TT virus DNA and the CD4 cell count in patients infected with HIV. AIDS.

