# Supplementary material for: High-Density Lipoprotein Particles and Torque Teno Virus in Stable Outpatient Kidney Transplant Recipients
Source: Viruses. 2024 Jan 18;16(1):143. doi: 10.3390/v16010143 (PMC10818741; doi:10.3390/v16010143)
Supplement: Supplementary file 1 [file viruses-16-00143-s001.zip › viruses-2802241-supplementary.pdf]

Supplementary data

Supplementary Figure S1

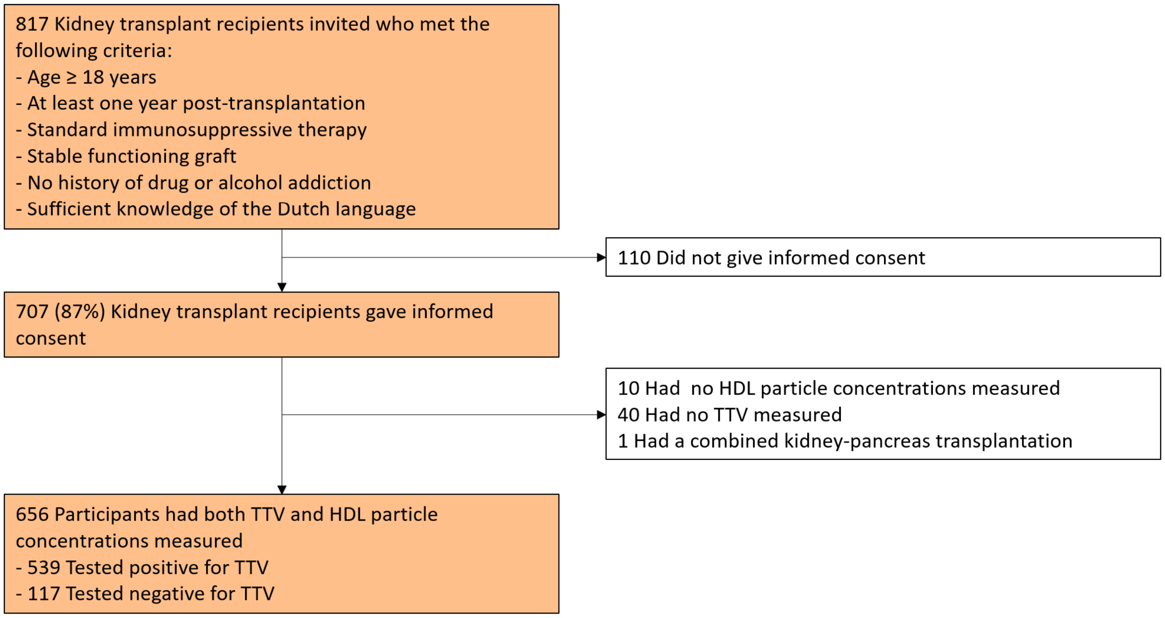

Supplementary Figure S1. flow chart illustrating the participant selection process for this article.

Supplementary Table S1

Supplementary Table S1. results of multivariable linear regression using. TTV load is the dependent variable. Continuous variables were standardized (st.β. with 95 % confidence intervals). Model 5 is the only model that includes data from both the non-smoking and the smoking TTV positive participants.

	Model 5 (R^2 = 0.12, n = 500)		
	St. β	95% CI st. β	P-value
Age of the recipient	0.11	0.02; 0.20	0.020
Female sex	-0.04	-0.23; 0.14	0.638
Smoking behaviour			
- Never smoked cigarettes	Reference		
- Stopped smoking cigarettes	-0.12	-0.31; 0.07	0.219
- Current cigarette smoking	-0.36	-0.64; -0.08	0.012
Average daily alcohol intake >10 grams	-0.12	-0.31; 0.08	0.238
Hemoglobin	-0.07	-0.16; 0.03	0.173
eGFR	-0.05	-0.15; 0.06	0.385
Age of the donor	-0.03	-0.13; 0.08	0.616
Allograft vintage	-0.10	-0.20; 0.01	0.064
Calcineurin inhibitor usage			
- Not using a calcineurin inhibitor	Reference		
- Using cyclosporin	0.32	0.12; 0.52	0.002
- Using tacrolimus	0.56	0.30; 0.82	<0.001
Total HDL particle concentration	-0.10	-0.19; -0.01	0.024

Supplementary Table S2

Supplementary Table S2. results of multivariable linear regression using. TTV load is the dependent variable. Continuous variables were standardized (st.β. with 95 % confidence intervals). Models 6 to 8 contain particle concentrations of the size-based HDL subspecies, model 9 contains all concentrations of the three size-based HDL subspecies. Abbreviations: sHDL, small HDL, mHDL, medium HDL; lHDL, large HDL.

	Model 6 (R^2 = 0.11, n = 440)			Model 7 (R^2 = 0.10, n = 440)			Model 8 (R^2 = 0.11, n = 440)			Model 9 (R^2 = 0.12, n = 440)		
	St. β	95% CI st. β	P-value	St. β	95% CI st. β	P-value	St. β	95% CI st. β	P-value	St. β	95% CI st. β	P-value
Age of the recipient	0.07	-0.02; 0.17	0.121	0.08	-0.01; 0.18	0.084	0.10	0.00; 0.19	0.048	0.08	-0.02; 0.17	0.112
Female sex	-0.04	-0.23; 0.15	0.689	-0.03	-0.23; 0.16	0.741	0.00	-0.20; 0.20	0.982	0.03	-0.17; 0.23	0.773
Average daily alcohol intake >10 grams	-0.18	-0.39; 0.02	0.076	-0.2	-0.40; 0.01	0.059	-0.20	-0.40; 0.01	0.060	-0.15	-0.36; 0.06	0.159
Hemoglobin	-0.05	-0.16; 0.05	0.321	-0.06	-0.17; 0.04	0.248	-0.07	-0.18; 0.03	0.170	-0.06	-0.16; 0.05	0.291
eGFR	-0.06	-0.17; 0.04	0.238	-0.07	-0.18; 0.04	0.191	-0.06	-0.17; 0.05	0.274	-0.04	-0.15; 0.07	0.431
Age of the donor	-0.03	-0.14; 0.09	0.657	-0.02	-0.13; 0.09	0.707	-0.02	-0.13; 0.09	0.684	-0.02	-0.13; 0.09	0.672
Allograft vintage	-0.09	-0.20; 0.02	0.103	-0.08	-0.20; 0.03	0.141	-0.09	-0.20; 0.03	0.129	-0.08	-0.20; 0.03	0.139
Calcineurin inhibitor usage												
- Not using a calcineurin inhibitor	Reference			Reference			Reference			Reference		
- Using cyclosporin	0.28	0.07; 0.50	0.010	0.32	0.10; 0.54	0.005	0.31	0.10; 0.53	0.004	0.31	0.09; 0.53	0.005
- Using tacrolimus	0.60	0.32; 0.89	<0.001	0.62	0.34; 0.90	<0.001	0.64	0.36; 0.92	<0.001	0.61	0.32; 0.89	<0.001
sHDL concentration	-0.10	-0.19; 0.00	0.044							-0.12	-0.22; -0.02	0.017
mHDL concentration				-0.02	-0.12; 0.08	0.682				-0.06	-0.17; 0.04	0.220
lHDL concentration							-0.07	-0.16; 0.03	0.182	-0.07	-0.17; 0.03	0.163

Supplementary Table S3

Supplementary Table S3. results of multivariable linear regression using. TTV load is the dependent variable. Continuous variables were standardized (st.β. with 95 % confidence intervals). Models 10 to 12 contain tHDL particle concentration and proportions of the subspecies. Model 13 is the sensitivity analysis wherein the participants with the 2.5% highest and 2.5% lowest TTV load and also the participants with the 2.5% highest and 2.5% lowest tHDL concentration were excluded. Abbreviations: sHDL, small HDL, mHDL, medium HDL; lHDL, large HDL.

	Model 10 (R^2 = 0.12, n = 440)			Model 11 (R^2 = 0.12, n = 440)			Model 12 (R^2 = 0.12, n = 440)			Model 13 (R^2 = 0.09, n = 416)		
	St. β	95% CI st. β	P-value	St. β	95% CI st. β	P-value	St. β	95% CI st. β	P-value	St. β	95% CI st. β	P-value
Age of the recipient	0.08	-0.02; 0.17	0.109	0.08	-0.02; 0.17	0.109	0.07	-0.02; 0.17	0.129	0.07	-0.03; 0.17	0.154
Female sex	0.03	-0.18; 0.23	0.789	0.03	-0.17; 0.23	0.754	0.03	-0.18; 0.23	0.801	-0.01	-0.23; 0.20	0.904
Average daily alcohol intake >10 grams	-0.15	-0.36; 0.06	0.156	-0.15	-0.35; 0.06	0.164	-0.15	-0.35; 0.06	0.160	-0.13	-0.35; 0.09	0.244
Hemoglobin	-0.06	-0.16; 0.05	0.294	-0.06	-0.16; 0.05	0.295	-0.05	-0.16; 0.05	0.321	-0.10	-0.21; 0.01	0.084
eGFR	-0.04	-0.15; 0.07	0.424	-0.04	-0.15; 0.07	0.429	-0.05	-0.16; 0.06	0.416	0.01	-0.10; 0.12	0.870
Age of the donor	-0.02	-0.13; 0.09	0.676	-0.02	-0.13; 0.09	0.682	-0.02	-0.13; 0.09	0.679	-0.05	-0.17; 0.06	0.363
Allograft vintage	-0.08	-0.19; 0.03	0.138	-0.08	-0.19; 0.03	0.144	-0.08	-0.19; 0.03	0.138	-0.11	-0.22; 0.01	0.072
Calcineurin inhibitor usage												
- Not using a calcineurin inhibitor	Reference			Reference			Reference			Reference		
- Using cyclosporin	0.31	0.09; 0.53	0.005	0.31	0.10; 0.53	0.005	0.31	0.10; 0.52	0.004	0.27	0.04; 0.50	0.020
- Using tacrolimus	0.60	0.32; 0.88	<0.001	0.60	0.32; 0.88	<0.001	0.60	0.32; 0.88	<0.001	0.42	0.12; 0.72	0.007
Total HDL particle concentration	-0.14	-0.23; -0.04	0.006	-0.14	-0.23; -0.04	0.006	-0.14	-0.24; -0.04	0.006	-0.14	-0.24;-0.04	0.008
sHDL proportion	0.00	-0.10; 0.09	0.934									
mHDL proportion				-0.01	-0.10; 0.09	0.910						
lHDL proportion							0.01	-0.09; 0.11	0.820			

STROBE Statement- checklist for cross-sectional studies

	Item No	Recommendation	Page No
Title and abstract	1	(*a*) Indicate the study's design with a commonly used term in the title or the abstract	Abstract
		(*b*) Provide in the abstract an informative and balanced summary of what was done and what was found	Abstract
Introduction			
Background/rationale	2	Explain the scientific background and rationale for the investigation being reported	Introduction paragraph 2 trough 5
Objectives	3	State specific objectives, including any prespecified hypotheses	Introduction paragraph 2 and paragraph 6
Methods			
Study design	4	Present key elements of study design early in the paper	Methods – Study population
Setting	5	Describe the setting, locations, and relevant dates, including periods of recruitment, exposure, follow-up, and data collection	Methods – Study population and Collection of data
Participants	6	(*a*) Give the eligibility criteria, and the sources and methods of selection of participants	Methods – Study population
Variables	7	Clearly define all outcomes, exposures, predictors, potential confounders, and effect modifiers. Give diagnostic criteria, if applicable	Table 1
Data sources/ measurement	8	For each variable of interest, give sources of data and details of methods of assessment (measurement). Describe comparability of assessment methods if there is more than one group	Methods – Collection of data paragraph 1 through 3
Bias	9	Describe any efforts to address potential sources of bias	Methods – Statistical analysis paragraph 3
Study size	10	Explain how the study size was arrived at	Supplementary Figure 1 and Methods – Study Population
Quantitative variables	11	Explain how quantitative variables were handled in the analyses. If applicable, describe which groupings were chosen and why	Methods – Statistical analysis paragraph 1 trough 3
Statistical methods	12	(*a*) Describe all statistical methods, including those used to control for confounding	Methods – Statistical analysis paragraph 1 trough 3
		(*b*) Describe any methods used to examine subgroups and interactions	Methods – Statistical analysis paragraph 2 and paragraph 3
		(*c*) Explain how missing data were addressed	Methods – Statistical analysis paragraph 2 and Footnote Table 1
		(*d*) If applicable, describe analytical methods taking account of sampling strategy	Not applicable
		(*e*) Describe any sensitivity analyses	Methods – Statistical analysis paragraph 3
Results			
Participants	13	(a) Report numbers of individuals at each stage of study—eg numbers potentially eligible, examined for eligibility, confirmed eligible, included in the study, completing follow-up, and analysed	Methods – Study population and Supplementary Figure 1
		(b) Give reasons for non-participation at each stage	Methods – Study population and Supplementary Figure 1
		(c) Consider use of a flow diagram	Supplementary Figure 1
Descriptive data	14	(a) Give characteristics of study participants (eg demographic, clinical, social) and information on exposures and potential confounders	Table 1 and Results – Primary outcomes paragraph 1
		(b) Indicate number of participants with missing data for each variable of interest	Footnote Table 1
Outcome data	15	Report numbers of outcome events or summary measures	Table 1 and Results – Primary outcomes paragraph 1

Main results	16	(*a*) Give unadjusted estimates and, if applicable, confounder-adjusted estimates and their precision (eg, 95% confidence interval). Make clear which confounders were adjusted for and why they were included	Table 1, Table 2 and Supplementary Table 1
		(*b*) Report category boundaries when continuous variables were categorized	Methods – Collection of data paragraph 2
		(*c*) If relevant, consider translating estimates of relative risk into absolute risk for a meaningful time period	Not applicable
Other analyses	17	Report other analyses done—eg analyses of subgroups and interactions, and sensitivity analyses	Methods – Statistical analysis paragraph 3
Discussion			
Key results	18	Summarise key results with reference to study objectives	Discussion paragraph 1
Limitations	19	Discuss limitations of the study, taking into account sources of potential bias or imprecision. Discuss both direction and magnitude of any potential bias	Discussion paragraph 3 and paragraph 7
Interpretation	20	Give a cautious overall interpretation of results considering objectives, limitations, multiplicity of analyses, results from similar studies, and other relevant evidence	Discussion paragraph 2
Generalisability	21	Discuss the generalisability (external validity) of the study results	Discussion paragraph 4 trough 6
Other information			
Funding	22	Give the source of funding and the role of the funders for the present study and, if applicable, for the original study on which the present article is based	Funding and Acknowledgments
